# Genetic variation of the gene coding for microRNA-204 (miR-204) is a risk factor in acute myeloid leukaemia

**DOI:** 10.1186/s12885-018-4045-y

**Published:** 2018-01-30

**Authors:** Aleksandra Butrym, Piotr Łacina, Kazimierz Kuliczkowski, Katarzyna Bogunia-Kubik, Grzegorz Mazur

**Affiliations:** 1 Department of Internal and Occupational Diseases, Hypertension and Clinical Oncology, Wroclaw Medical University, Wrocław, Poland; 2 Laboratory of Clinical Immunogenetics and Pharmacogenetics, Hirszfeld Institute of Immunology and Experimental Therapy, Polish Academy of Sciences, Wrocław, Poland; 3 Department of Haematology, Blood Neoplasms and Bone Marrow Transplantation, Wroclaw Medical University, Wrocław, Poland

**Keywords:** microRNA, miR-204, Polymorphism, Acute myeloid leukemia, Disease susceptibility, Survival

## Abstract

**Background:**

MicroRNAs (miRNAs or miRs) are small molecules known to be involved in post-transcriptional gene expression. Many of them have been shown to influence risk for various diseases. Recent studies suggest that lower expression of *miR-204*, a gene coding for miRNA-204, is correlated with shorter survival in patients with acute myeloid leukaemia (AML). This observation prompted us to analyse the effect of two polymorphisms of the *miR-204* gene, one in the upstream flanking region (rs718447 A > G) and the other inside the gene itself (rs112062096 A > G), both also in intron 3 of the TRPM3 gene.

**Methods:**

The study was conducted on DNA samples isolated from AML patients (*n* = 95) and healthy individuals (*n* = 148), who were genotyped using the Light SNiP assays.

**Results:**

The *miR-204* rs718447 *GG* homozygosity was found to constitute a risk factor associated with susceptibility to AML (73/95 vs 92/148, AML patients vs healthy controls, OR = 2.020, *p* = 0.017). Additionally, this genotype was more frequent in patients with subtypes M0-M1 in the French-American-British (FAB) classification as compared to patients with subtypes M2-M7 (23/25 vs 39/57, *p* = 0.026). We also found that presence of allele *A* was linked to longer survival of AML patients.

**Conclusions:**

Our results show that polymorphism in *miR-204* flanking region may constitute a risk and prognostic factor in AML.

## Background

Acute myeloid leukaemia (AML) is a very aggressive and heterogeneous haematological malignancy. It results from many complex genetic and epigenetic dysregulations that influence haematopoietic stem cell differentiation and maturation. All the aforementioned alterations lead to uncontrolled proliferation of the myeloblasts in the bone marrow [1]. The prognosis of AML patients remains poor. Although significant progress has already been made in the diagnostic and therapeutic process, new factors are still being investigated as potential risk factors for AML development and clinical outcome.

microRNAs (miRs) are small, non-coding RNAs that can act as epigenetic regulators of gene expression and affect signalling pathways. Some of them can enhance tumour progression and metastasis [2]. Several miRs have been described as risk factors in acute myeloid leukaemia patients [3, 4]. Several studies have shown the importance of single nucleotide polymorphisms (SNPs) for the risk of cancer development. Such SNPs are located either in genes involved in microRNA biogenesis or inside microRNA binding sites (MBS) at the target mRNA [5, 6].

In the present study, we examined two *miR-204* SNPs. The *miR-204* gene is located in chromosome 9, in locus 9q21.12. It is situated inside the intronic sequence of another gene, *TRPM3*, between exons 6 and 7. The rs112062096 variation is found within the *miR-204* gene itself, while the rs718447 variation is located in a flanking region of the gene.

The rs718447 SNP does not result in any difference in the miRNA-204 sequence, although it may influence expression of the gene, and different expression is associated with outcome of the disease [7]. As the *miR-204* gene lies inside an intron in the *TRPM3* gene, it is possible that rs718447 can affect AML through change in *TRPM3* expression. However, to the best of our knowledge, there is no known literature indicating any association of TRPM3 with AML.

## Methods

### Patients characteristics

Ninety-five patients (aged 22–90, median age 61; 56 males and 39 females) with newly diagnosed AML were included in the study. Blood samples for *miR-204* polymorphism genotyping were collected before induction chemotherapy. 7 patients had AML M0, 34 had M1, 29 had M2, 14 had M4 and 11 had M5 according to AML French-American-British (FAB) classification. The range for lactate dehydrogenase (LDH) was 108–4565 U/l (median equalled 369 U/l). Analysis of FLT3 mutation was performed in 60 patients (13 were positive for FLT3/ITD and 47 were negative). Median survival was 3 months (range 0–55). We also used samples of 148 healthy persons as a control group.

### Genotyping

DNA was extracted from samples of peripheral blood taken on EDTA using Maxwell 16 Blood DNA Purification Kit (Promega Corp., USA) or silica membranes (Qiagen, Germany) following recommendations of the manufacturers. Both SNPs (rs718447 A > G and rs112062096 A > G) of the *miR-204* gene were determined by the LightSNiP assay (TIB-MolBiol, Berlin, Germany) on a LightCycler 480 Real-Time PCR system (Roche Applied Science, Mannheim, Germany).

### Statistical analysis

Fisher’s exact test was used to test the null hypothesis that there is no difference between allele and genotype frequencies between patients and controls. The odd’s ratio (OR) was calculated by Haldane’s modification of Woolf’s method and the significance of its deviation from unity was estimated by Fisher’s exact test. The nonparametric Man-Whitney test was used to test the null hypothesis that the survival values are equally large for bearers of either of the possible genotypes. Probability values < 0.05 were considered statistically significant, and those between 0.05 and 0.10 as indicative of a trend. All genotypes were tested for deviations from Hardy-Weinberg equilibrium using the chi-squared test.

## Results

### Distribution of *miR-204* rs718447 and rs112062096 alleles and genotypes in patients and controls

**Fig. 1:**
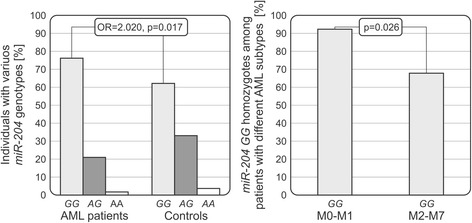
Associations of miR-204 SNP with susceptibility and progression of acute myeloid leukaemia. The left panel shows distribution of the *miR-204* rs718447 alleles and genotypes in AML patients and in the control group. The right panel presents the relationship between *miR-204* rs718447 *GG* genotype and FAB subtype of the disease

All of patients and controls were genotyped for both SNPs. For *miR-204* rs718447, the genotype frequencies were as follows: 2.11% *AA*, 21.05% *AG,* 76.84% *GG* for AML patients and 4.73% *AA*, 33.11% *AG*, 62.16% *GG* for healthy controls; the frequencies observed for rs718447 were in accordance with Hardy-Weinberg equilibrium (*P* = 0.701 for patients and *P* = 0.881 for controls). The *miR-204* rs718447 allelic frequencies were 0.126 *A* and 0.874 *G* for patients and 0.213 *A* and 0.787 *G* for controls. In *miR-204* rs112062096, only the *AA* genotype was detected (Fig. 1).

### Associations of *miR-204* rs718447 with risk and subtype

The rs718447 SNP demonstrated a significant difference between cases and controls. The rs718447 *GG* homozygous genotype was seen more frequently in AML patients than in healthy individuals (73/95 vs 92/148, *P* = 0.017), with the odds ratio (OR = 2.020, 95% CI 1.130**–**3.611).

We also observed a difference between patients with different subtypes of AML (according to the FAB classification). Here, patients with subtypes M0-M1 (undifferentiated AML and AML with minimal maturation) were more often rs718447 *GG* homozygotes more often than patients with subtypes M2-M7 (23/25 vs 39/57, *P* = 0.026).

### Associations of *miR-204* rs718447 with prognostic factors

**Fig. 2:**
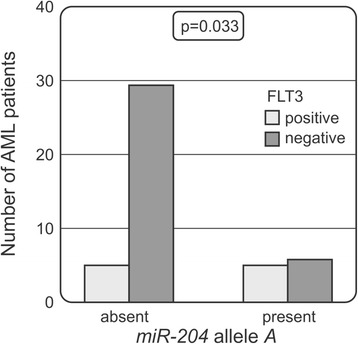
Relationship between *miR-204* rs718447 and FLT3/ITD mutation status

We also looked at FLT3 mutational status in AML patients and found that patients with non-mutated FLT3 were less frequently carriers of allele *A* (*P* = 0.033, Fig. 2). The group of AML patients with mutated FLT3 was quite small and statistical analysis was difficult to perform, but 45% of patients with mutated FLT3 had the allele *A* present.

Patients were also checked for associations with prognostic parameters. Analysis of white blood cell count (WBC) showed no statistically significant difference between patients with WBC above the normal range and those with WBC below the upper limit of the range (10,000 cells/μL; *P* = 0.427). However, when we investigated only patients with WBC higher than normal, it turned out that those with WBC higher than the median value for this population (38,230 cells/μL) were more often *GG* homozygotes than those with WBC lower than the median (26/30 vs 17/29, *P* = 0.020). This association was also significant when all patients (also those with WBC in or below normal range) were included (50/59 vs 17/29, *P* = 0.015).

We also analysed association of the *miR-204* polymorphism with lactate dehydrogenase (LDH) concentration in blood. Similarly to WBC, no statistically significant difference between patients with levels above the normal range and those with levels below the upper limit of the range (220 U/L) was detected (*P* = 1.000). When we calculated the median of LDH concentration of our patients (369 U/L) and used it to divide all our patients into two roughly equal groups, we observed that the *GG* homozygosity is more frequent in patients with LDH concentrations lower than the median (37/43 vs 26/41, *P* = 0.023). Furthermore, when we analysed only patients above the normal LDH range of 220 U/L, while still using 369 U/L as a separator number, we observed that the aforementioned correlation is even stronger (25/27 vs 26/41, *P* = 0.009).

### Associations of *miR-204* rs718447 and survival

**Fig. 3:**
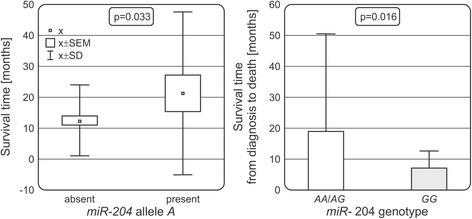
Associations of miR-204 SNP with survival of AML patients. Relationship between *miR-204* rs718447 *A* allele and survival in AML patients is presented in the left panel, while survival of AML patients (only subjects who have died included) in relation to rs718447 genotype is in the right panel

We compared survival time, i.e. time from diagnosis to death (or end of observation, in patients who are still alive), between patients with different *miR-204* rs718447 genotypes and observed that the *GG* homozygotes seem to have shorter survival time than carriers of other genotypes. The presence of the allele *A* had a significant effect on AML patients’ survival. Patients carrying this *A* allele had longer survival than patients without allele *A* (21.3 months vs 12.4 months, *P* = 0.033 Fig. 3). Moreover, analysis in a subgroup of patients in whom death event occurred, homozygous *GG* genotype was correlated with shorter survival (*P* = 0.016, Fig. 3).

## Discussion

Recently, expression of *miR-204* was shown to be down-regulated in AML patients [7]. In our present study, we found the rs718447 SNP in the flanking region of the *miR-204* gene is associated with risk, subtype and survival of AML patients. microRNAs are potent molecules affecting numerous pathways and processes [8]. In this study, we focused on miR-204, which is known to be associated with many diseases, among them e.g. endometrial cancer, neuroblastoma, pancreatic cancer, gastric cancer and acute myeloid leukaemia [9–12]. We examined two SNPs inside the *miR-204* gene and its flanking region – rs112062096 and rs718447 – both of which have never been studied before in any disease. We looked into potential associations with risk, course of disease, outcome of treatment and other factors associated with AML.

The rs112062096 *G* allele was completely absent in our study groups. To the best of our knowledge, this has also never been described in any paper before. The entry for this SNP in the dbSNP NCBI database is based entirely on a population from Northern Kalahari desert (a region straddling the Namibia-Botswana border in southern Africa), therefore we suggest that this genetic variation is either mostly restricted to this region in Africa and is rare elsewhere or is entirely absent in Caucasian populations. Further studies on other populations would be required to fully validate either of these hypotheses.

As for the rs718447 variation, we have found that the rs718447 *GG* genotype is associated with increased risk for AML. Since it was shown by our group before that decreased miRNA-204 expression is correlated with risk for AML [7], it might be possible that the *GG* homozygosity induces lower expression levels than the *AG* and *AA* genotypes. To assess the possible correlation between the miRNA-204 levels and rs718447 genotype in AML patients we took into account the results of our previous study [7]. However, we were able to include only 30 patients in the preliminary analysis, and we did not find any statistically significant correlation. It is possible that either the correlation would be visible in an experiment with a larger number of patients or that a different mechanism causes the higher risk in patients with the rs718447 *GG* genotype.

The most important of our findings was the correlation between presence of *the wild-type* allele *A* and longer survival in AML patients. It seems that allele *A* has a protective role in AML patients.

We also found that the *GG* genotype occurs more often in patients with LDH levels higher than 369 U/L (the median for our group). The 369 U/L cut-off point is much higher than the normal range of app. 220 U/L, however it has been shown earlier that LDH levels higher than 300 U/L, which is also well above the normal range, are associated with worse survival and disease progression [13, 14].

The mechanism behind the reported correlations is unknown, although the SNP Function Prediction tool available at the National Institute of Environmental Health Sciences website shows that the rs718447 SNP is predicted to be located in a transcription factor binding site [15]. It can be hypothesized that rs718447 variants cause differential miR-204 expression. The mode of action of all microRNAs is by impeding expression of other genes and as such, the observed difference in survival might be a result of varying miR-204 expression affecting expression of another, unknown gene.

Potential targets of miR-204 have been described in various diseases, although not in AML. A study on gastric cancer indicated that Bcl-2, a protein affecting apoptosis, might be the target of miR-204. In silico analysis showed that *BCL2* 3’UTR contains a functional miR-204-binding site and an experiment with mutated *BCL2* 3’UTR showed confirmed that miR-204 targets Bcl-2 and its downregulation results in aberrant Bcl-2 expression in a gastric adenocarcinoma cell line [16]. This pro-apoptotic action of miR-204 through BCL2 targeting was also shown in prostate cancer cells [17]. Another study confirmed that miR-204 binds to *BCL2* mRNA in a neuroblastoma cell line and showed that it may also bind to *NTRK2* (neurotrophic receptor tyrosine kinase 2) [10]. In contrast to those reports, a study on endometrial cancer cells showed that miR-204 does not affect apoptosis in those cells but instead affects cell migration and invasion. The same study identified *FOXC1* as a direct target of miR-204 and suggested that they might be involved in progression and metastasis in endometrial cancer [9]. An association with *FOXC1*, miR-204 and metastasis was also recently shown for laryngeal squamous cell carcinoma [18]. A study on glioblastoma cell lines also found miR-204 to affect migration and invasion, although due to interaction with *ATF2* mRNA [19]. Similarly, a study on cervical cancer showed that miR-204 targeting another gene, *EPHB2*, affects progression, invasion and migration [20]. Interestingly, it has recently been found in T-cell acute lymphoblastic leukaemia, a haematological disease, that miR-204 binds to SOX4 to inhibit proliferation, migration and invasion [21]. A similar finding was shown for renal cell carcinoma and gastric cancer [22, 23].

It is impossible to assess which of the aforementioned proteins being targeted by miR-204 is responsible for the SNP effects on survival described in the present study. It is, however, worth noting that BCL2 was shown earlier to affect AML treatment and, together with FLT3 status, correlated with reduced disease-free survival [24]. This effect is believed to be due to altered BCL2 expression affecting apoptosis and multi-drug resistance [25]. Likewise, FOXC1 expression was observed to be higher in AML haematopoietic and progenitor stem cells than in normal ones; it was also noticed that FOXC1-high cases exhibited worse survival than FOXC1-low ones. FOXC1 is likely to cause this effect by blocking monocyte/macrophage differentiation [26]. SOX4, a transcription factor involved in cell differentiation and cell survival, has also been shown to correlate with AML prognosis, being associated with overall and disease-free survival in AML patients, irrespective of other clinical parameters [27]. It is thus possible that changes in expression of one of the described proteins are ultimately responsible for SNP associations observed in our study. Naturally, further research would be required to prove these observations and to indicate, which proteins are responsible specifically.

## Conclusions

To the best of our knowledge, this is the first report of an association between *miR-204* polymorphism and acute myeloid leukaemia. We found the rs718447 *GG* genotype to be a risk factor for AML development and worse prognosis. As our study group is relatively small, these results need to be confirmed on a larger cohort of AML patients.

## Abbreviations

AML: Acute myeloid leukaemia
FAB: French-American-British
LDH: Lactate dehydrogenase
MAF: micro allele frequency
MBS: Microrna binding site
miR: microRNA
SNPs: Single nucleotide polymorphism
WBC: White blood cells
